# Higher serum zinc micronutrient levels are associated with reduced susceptibility to Group B *Streptococcus* rectovaginal colonisation in pregnant women

**DOI:** 10.1371/journal.pone.0344689

**Published:** 2026-03-12

**Authors:** Nisha Dhar, Jennifer Gaddy, David Michael Aronoff, Kalavati Channa, Shabir Ahmed Madhi, Gaurav Kwatra

**Affiliations:** 1 South African Medical Research Council Vaccines and Infectious Diseases Analytics Research Unit, Faculty of Health Sciences, University of the Witwatersrand, Johannesburg, South Africa; 2 Division of Infectious Diseases, Cincinnati Children’s Hospital Medical Center, Cincinnati, Ohio, United States of America; 3 Division of Infectious Diseases, Department of Medicine, Vanderbilt University Medical Center, Nashville, Tennessee, United States of America; 4 Department of Medicine, Indiana University School of Medicine, Indianapolis, Indiana, United States of America; 5 Lancet Laboratories, Department of Analytical Chemistry, Johannesburg, South Africa; 6 Wits Infectious Diseases and Oncology Research Institute, Faculty of Health Sciences, University of the Witwatersrand, Johannesburg, South Africa; 7 Department of Paediatrics, University of Cincinnati College of Medicine, Cincinnati, Ohio, United States of America; 8 Department of Clinical Microbiology, Christian Medical College, Vellore, India; CSSRI: Central Soil Salinity Research Institute, INDIA

## Abstract

**Background:**

Maternal recto-vaginal colonisation by Group B *Streptococcus* (GBS) is a major risk factor for severe invasive GBS disease in newborns. Zinc is a key micronutrient known to promote defence against bacterial infections. We hypothesized that adequate zinc micronutrient levels in pregnant women would negatively affect GBS colonisation and persistence during pregnancy.

**Objective:**

To determine the association between serum zinc levels and risk of recto-vaginal GBS colonisation acquisition in pregnant women, as well as the potential for clearance of colonisation later in pregnancy.

**Methods:**

Zinc concentrations were analysed in serum samples from women who acquired rectovaginal GBS colonisation and from women who cleared GBS colonisation between 20 weeks and 37–40 weeks of gestational age. Zinc concentration at 20–25 weeks and 37–40 weeks gestational age was measured using inductively coupled plasma mass spectrometry.

**Results:**

Higher baseline serum zinc concentration was associated with a lower risk of new GBS acquisition [Odds ratio (OR) 0.15, p = 0.001]. Zinc geometric mean concentration was higher in women who were persistently un-colonised by GBS compared with those with a new acquisition of GBS (20.18 vs 13.68 µmol/L; p = 0.03). The lowest zinc threshold ≥15 µmol/L was associated with significantly reduced odds of new GBS acquisition (27.2% in new acquisition vs 40.5% in persistently un-colonised; OR 0.55; 95%CI 0.31–0.96; p = 0.03). An association was also evident between 15–20 umol/L serum zinc levels and greater odds of GBS colonisation clearance.

**Conclusions:**

This study demonstrates a potential role of serum zinc nutrition in the reduced risk of recto-vaginal GBS colonisation during pregnancy.

## Introduction

Transmission of Group B *Streptococcus* (GBS) to a fetus from vaginally and/or rectally colonised women, through ascending intrauterine infection or as the baby passes through the birth canal during parturition, is a major risk factor associated with severe early-onset invasive GBS disease (EOD) in the first seven days of newborn life [1,2]. In 2020, approximately 394,000 cases of invasive GBS disease occurred in infants globally, resulting in an estimated 90,000 deaths [3]. Furthermore, ascending GBS infections from women with recto-vaginal colonisation contribute to at least 3.5 million preterm births and 57,000 stillbirths worldwide per year [4]. Globally, approximately 18% of women are colonised with GBS, with the highest prevalence (41.1%) reported in Mali [1,5].The current intrapartum antibiotic prophylaxis (IAP) strategy given to GBS-colonised women during labour is logistically difficult to implement in low- and middle- income countries (LMICs) [2,6]. Furthermore, IAP is unlikely to reduce the risks of GBS-associated preterm and stillbirths. Maternal immunization with a GBS vaccine is a promising strategy to prevent invasive disease in infants; however, licensure is challenged by difficulties in evaluating vaccine efficacy, as well as considerations of cost-effectiveness and public acceptance [7]. Consequently, there is a need to explore other interventions to reduce the risk of maternal recto-vaginal GBS colonisation and invasive disease in newborns.

Zinc is one of the essential micronutrients that determine immunity against bacterial infections in mother-infant dyads [8]. Supplementation with zinc reduces the risk of mucosal bacterial infections in humans [9]. Zinc is involved in host immunity through inhibition of biofilm formation required for adherence, persistence and colonisation on epithelial mucosa by various bacterial pathogens including *Streptococcus* species [10,11]. Low zinc availability results in increased bacterial biofilm formation *in vitro*, whereas zinc supplementation eradicates biofilm communities [10,12]. Furthermore, zinc promotes defence against bacterial infections through maintaining the host epithelial barrier integrity and function, activation of immune cells, pro-inflammatory responses, signalling and nutritional immunity via micronutrient sequestration or intoxication [13,14]. Our previous study demonstrated that increasing zinc concentrations significantly inhibited the growth of GBS strains across a diverse panel of colonising and invasive isolates, varying by capsular serotype, sequence type, isolation source, and clinical presentation [15]. We therefore hypothesized that adequate zinc micronutrient levels among pregnant women would negatively affect GBS colonisation and persistence during pregnancy. This study investigated the association of serum zinc level at 20–25 weeks of gestational age in pregnant women and risk of recto-vaginal new acquisition of GBS; as well as the potential for clearance of GBS colonisation during the latter part of pregnancy.

## Materials and methods

### Study design

We investigated stored serum samples from a previously enrolled cohort of pregnant women at four antenatal clinics in Soweto, Johannesburg from 2010 to 2011 [16]. Briefly, the study cohort consisted of 661 pregnant women who attended routine community antenatal clinics in Soweto, South Africa. Inclusion criteria included pregnant women aged 18−45 years at 20−25 weeks gestational age, who were not infected with human immunodeficiency virus. Exclusion criteria included women with acute illness or a symptomatic vaginal discharge, those who had received antibiotic treatment in the previous two weeks, and/or those with a known or suspected condition for which clinical vaginal examination was contraindicated. As part of the study‑specific research protocol, pregnant women were enrolled at 20–25 weeks of gestation (visit-1) and subsequently followed at 5–6-week intervals until 37 + weeks of gestation (visit-4, prior to delivery).

At each visit, participants were considered positive for GBS colonisation if GBS was identified from either vaginal, rectal, or both swabs and negative if GBS was not detected from both the swabs as described previously [16]. Whole blood samples were collected from pregnant women at 20–25 weeks and 37 + weeks gestation. Serum was extracted from the blood within 4 hours of collection and stored at −70 degree Celsius until analysis. Studies have shown that concentrations of zinc in blood and/or plasma specimens remain stable during long term storage at freezing temperatures [17]. We accessed stored participant samples and demographic information between March 1, 2019, and May 31, 2019. Of the 661 enrolled participants, 507 completed four visits, of those recto-vaginal GBS colonisation status and serum samples were available for 466 participants for the current study. The authors had no access to personally identifiable information of individual participants during or after data collection.

### Serum zinc analysis

Zinc concentration in serum was tested at visit-1 and visit-4. Zinc level estimation in serum samples was performed at Lancet laboratories, Johannesburg, South Africa using inductively coupled plasma mass spectrometry according to the laboratory’s standard protocol. Briefly, samples were diluted 20-fold with a serum diluent. Appropriate internal standards and reference controls were used for the generation of a calibration curve with r-squared value greater than 0.999. Zinc concentration was measured in triplicates, and the average value was reported in µmol/L. The limit of quantitation was 0.005 µmol/l. Zinc deficiency was considered present in women who had serum zinc concentration <7.6 µmol/L, which is the lower cut off for normal zinc concentration observed in pregnant women during second and third trimester [18].

### Statistical analyses

Based on the recto-vaginal GBS colonisation status, women were categorized into four groups: persistently un-colonised, new acquisition, colonisation clearance and persistently colonised. The “new acquisition” group was defined as participants who were negative for GBS at visit-1 and subsequently acquired GBS in one of the following visits; and those not colonised with GBS at any of the visits were categorized as “persistently un-colonised”. Participants who were GBS positive at visit-1 but cleared GBS colonisation by the last visit (Visit-4) were classified as “colonisation clearance” group; and those who remained colonised throughout all study visits were grouped as “persistently colonised”.

Groups were compared using Mann-Whitney test for skewed data sets or the Student unpaired t- test for data sets with Gaussian distribution. The association between serum zinc concentration at visit-1 and GBS colonisation status was evaluated using a univariate logistic regression analysis, which reported the odds ratio (OR) with logarithmic zinc concentration as a covariate. The visit-1 and visit-4 zinc concentrations were compared using Wilcoxon matched-pairs signed-rank test. For all analyses, frequency with percentages, p values, geometric mean concentrations (GMCs) with 95% confidence interval (95% Cl), or median with inter-quartile range, as appropriate, were reported. For categorical variables, groups were compared using the Chi-square test and ORs were reported. Reverse cumulative plots were constructed and threshold concentrations were determined. Data were analyzed using GraphPad Prism version 7.04 software (GraphPad Software, San Diego, California) and Stata version 13 software (StataCorp, College Station, Texas). For all analyses, a p value <0.05 was considered statistically significant.

### Power analysis

For the current study, 466 participants who completed all four visits and for whom recto-vaginal GBS colonisation status and serum samples were available were included. Among the women not colonised with GBS at visit-1, 81 women acquired GBS during the course of the study, whereas 242 remained persistently un-colonised resulting in a new acquisition and un-colonised ratio of 1:3. Based on the difference in GMC’s and standard deviations among the two groups, this sample size yielded a power of above 90%. Similarly, among women who were GBS colonised initially, 76 women cleared colonisation by last visit, whereas 67 remained persistently colonised resulting in a GBS clearance and persistent colonisation ratio of 1:1.1. Based on the difference in GMC’s and standard deviation among the two groups, this sample size yielded a power of above 90%.

### Ethical approval

The primary study was approved by Human Research Ethics Committee (HREC), University of the Witwatersrand (Certificate# M090937). Written informed consent was obtained from all participating mothers. Additional approvals were obtained from University of the Witwatersrand, HREC to conduct this research on existing samples (Certificate# M1811137, M220562).

## Results

Among the women in whom GBS colonisation status and serum samples were available (n = 466), 69.3% (323/466) were not colonised with GBS at visit-1; among these women, 25% (81/323) acquired a new acquisition of GBS during one of the subsequent visits (S1 Figure in S1 File). Among 143 women who were GBS positive at visit-1, 53.1% (76/143) cleared GBS colonisation by visit-4 (S1 Figure in S1 File). There were no differences in the demographic characteristics between the “new acquisition” and “persistently un-colonised” groups; or between the “colonisation clearance” and “persistently colonised” groups (S1 Table in S1 File).

Zinc GMC at visit-1 was higher among the “persistently un-colonised” vs “new-acquisition” group (20.18 vs 13.68 µmol/L; p = 0.03); Table 1. In logistic regression analysis, higher zinc concentration was associated with significantly lower odds of new GBS acquisition (OR: 0.15, 95%CI 0.05–0.44; p = 0.001); Table 1. The strength of association between zinc concentration and reduced odds of new acquisitions tended to be stronger with increasing zinc concentrations (Table 1). The lowest zinc concentration threshold significantly associated with reduced odds of GBS acquisition was ≥ 15 µmol/L (40.5% in “persistently un-colonised” vs 27.2% in the “new-acquisition group”, OR: 0.55, 95%CI 0.31–0.96; p = 0.03); Table 1 and S3 Figure in S1 File. Zinc GMC’s between visit-1 and visit-4 were similar (p = 0.30; S2 Figure in S1 File). Zinc deficiency was present in 0.42% (2/466) and 1.7% (8/464) of women, at visit-1 and visit-4, respectively.

**Table 1:** Association of serum zinc concentration with Group B Streptococcus new acquisition in pregnant women.

Participants (n = 323)	New Acquisition (n = 81)	Persistently un-colonised (n = 242)	p value*	OR (95% CI); p value**
Zinc GMC at visit-1, umol/L (95% CI)	13.68 (12.59–14.87)	20.18 (17.99–22.64)	0.03	0.15 (0.05–0.44); 0.001
Zinc concentration at visit-1, umol/L	New Acquisition, Proportion (%)	Persistently un–colonised, Proportion (%)	OR (95% CI); p value^#^	
>=7.6	80/81 (98.8%)	241/242 (99.6%)	0.33 (0.017–6.38); 0.41	
>=10	71/81 (87.7%)	215/242 (88.8%)	0.89 (0.41–1.85); 0.77	
>=15	22/81 (27.2%)	98/242 (40.50%)	0.55 (0.31–0.96); 0.03	
>=17	16/81 (15.1%)	90/242 (84.9%)	0.42 (0.23–0.76); 0.004	
>=20	11/81 (13.6%)	80/242 (33.1%)	0.32 (0.16–0.63); 0.0007	
>=25	8/81 (9.9%)	66/242 (27.3%)	0.29 (0.14–0.63); 0.001	
>=50	1/81 (1.2%)	45/242 (18.6%)	0.05 (0.005–0.32); 0.0001	

Abbreviations: GMC: geometric mean concentration; 95% CI: 95% confidence interval; OR: Odds ratio.

* p value derived using Mann-Whitney test, ** p value derived using logistic regression analysis, ^#^ p value derived using Chi-square test.

The serum zinc GMC was higher at baseline in the “colonisation-clearance” (20.03 µmol/L; 95%CI 16.54–24.27) compared with the “persistently colonised” (16.45 µmol/L; 95%CI 13.32–20.31); p = 0.04 groups (Table 2). The overall zinc GMC was not significantly associated with the odds of GBS colonisation clearance. However, stratified analysis demonstrated a significant association for zinc concentrations within the range of 15–20 µmol/L and odds of GBS clearance. Specifically, participants with zinc levels ≥17 µmol/L exhibited 2.34-fold higher odds of GBS clearance (95% CI: 1.09–4.74; p = 0.02) in “colonisation clearance” group compared to those persistently colonised (38.2%, 29/76 vs. 20.9%, 14/67; see Table 2 and S4 Figure in S1 File).

**Table 2:** Association of serum zinc concentration with Group B Streptococcus colonisation clearance in pregnant women.

Participants (n = 143)	Colonisation clearance (n = 76)	Persistently colonised (n = 67)	p value *	OR (95% CI); p value **
Zinc GMC at visit-1, umol/L (95% CI)	20.03 (16.54–24.27)	16.45 (13.32–20.31)	0.04	1.92 (0.75–4.86); 0.17
Zinc concentration at visit-1, umol/L	Colonisation clearance, Proportion (%)	Persistently colonised, Proportion (%)	OR (95% CI); p value^#^	
>=7.6	76/76 (100%)	67/67 (100%)	Odds Ratio >0.9999	
>=10	68/76 (89.5%)	54/67 (80.6%)	2.0 (0.83 to 5.24); 0.13	
>=15	32/76 (42.1%)	18/67 (26.9%)	1.98 (0.99–3.91); 0.06	
>=17	29/76 (38.2%)	14/67 (20.9%)	2.34 (1.09–4.74); 0.02	
>=20	26/76 (34.2%)	13/67 (19.4%)	2.16 (1.03–4.60); 0.047	
>=25	24/76 (31.6%)	12/67 (17.9%)	2.12 (0.98 to 4.78); 0.06	
>=50	14/76 (18.4%)	10/67 (14.9%)	1.29 (0.52–3.19); 0.58	

Abbreviations: GMC: geometric mean concentration; 95% CI: 95% confidence interval; OR: Odds ratio.

* p value derived using Mann-Whitney test, ** p value derived using logistic regression analysis, ^#^ p value derived using Chi-square test.

## Discussion

Our investigation points to a potential role of serum zinc levels in reducing the risk of recto-vaginal GBS colonisation among pregnant women, with zinc concentration of ≥15 µmol/L associated with a 45% reduced risk of new GBS acquisition. A significant inhibitory effect of increasing zinc concentrations on the culture growth of a panel of colonising or invasive GBS strains was demonstrated in our previous study [15]. Our results confirm our hypothesis of a negative effect of zinc micronutrient levels on GBS colonisation during pregnancy. Several studies have reported a beneficial role of zinc nutrition in host defence against colonisation by various pathogenic bacteria on respiratory or enteric mucosa, lowering risks of bacterial infections substantially in humans [19]. In a double blinded, placebo controlled, randomized trial, zinc supplementation of mothers during pregnancy reduced the risks of acute diarrhoea [risk ratio (RR): 0.84] and dysentery (RR: 0.36) in infants for the first 6 months of their life [20]. In children, zinc supplementation is associated with 18%, 41% and more than 50% reduction in incidence of diarrhoea, pneumonia, and child mortality, respectively [9]. Furthermore, zinc supplementation reduced the risks of acute lower respiratory infection and pneumonia associated with nasopharyngeal *S. pneumoniae* colonisation in children [19].

Several studies have shown that zinc can restore bacterial infection–induced disruptions in epithelial permeability and maintain barrier integrity, facilitating bacterial clearance through non-specific barrier immune mechanisms [14]. In our study, the association observed between serum zinc concentrations of 15–20 umol/L and increased odds of GBS clearance indicates a potential therapeutic use of zinc in reducing GBS colonisation and the risk for invasive disease.

During the course of pregnancy, the average physiological requirement of zinc increases up to 38% in third trimester compared with pre-pregnancy, resulting in a decrease in serum zinc concentrations [21,22]. National surveys indicate that zinc deficiency is prevalent in more than 20% of women worldwide including around 21–84% of women of reproductive age in LMICs; and 46–76% of pregnant women in sub-Saharan Africa [23,24]. With changing food environments, food insecurity or food choices, a high proportion of pregnant women (82%) worldwide have inadequate dietary zinc intake [23]. Although, the prevalence of zinc deficiency in our cohort was < 2% with zinc levels similar at both measured timepoints, lower baseline zinc levels were strongly associated with a high risk of new GBS acquisition. This highlights the high susceptibility of population with inadequate zinc nutrition to GBS colonisation.

The IAP strategy given to GBS colonised women at 35–37 weeks of gestational age has reduced EOD in high-income countries, where implemented [6]. However, the adoption of this strategy has been challenging in LMICs due to the cost of the intervention, lack of resources and logistics challenges [25]. A GBS hexavalent capsular polysaccharide and protein-based vaccine is currently under phase 2 clinical trial, and there is presently no licensed GBS vaccine available [7]. Potential barriers to vaccine implementation include low disease incidence that constrains licensure based on efficacy endpoints; as well as regional heterogeneity in estimated vaccine cost-effectiveness, and anticipated vaccine acceptance [7]. Pre-conception and antenatal zinc supplementation could be a cost-effective and feasible strategy to support maternal zinc nutrition needs and potentially reduce risks of GBS colonisation during pregnancy and mitigate GBS associated adverse pregnancy outcomes, especially in LMICs. While our findings contribute valuable insights about maternal zinc nutrition, further investigational trials examining whether zinc supplementation pre-conception and in early pregnancy may reduce risk of maternal recto-vaginal GBS colonisation, and consequently invasive GBS disease in newborns are warranted.

### Limitations

This was a retrospective study that used archived serum samples for testing zinc. Serum zinc concentration as a biomarker for zinc status includes low sensitivity for marginal zinc status, diurnal variation, and fluctuation related to meals and time of day [18]. In addition, supplemental iron, acute and chronic infection and systemic inflammation may affect serum zinc concentrations [18]. However, serum zinc concentration is considered a useful biomarker for characterizing zinc status at a population level [26,27].

## Conclusions

This study demonstrates a strong association between serum zinc nutrition in pregnant women and the reduced risk of recto-vaginal GBS colonisation. While our findings contribute valuable insights into maternal zinc nutrition, further investigational trials examining whether zinc supplementation pre-conception and in early pregnancy may reduce the risk of maternal recto-vaginal GBS colonisation, and consequently invasive GBS disease in newborns are warranted.

## Supporting information

S1 File Supporting table and figures (S1 Table, and S1–S4 Figures). (DOCX)

S2 File Raw data for zinc concentrations and colonisation status. (XLSX)

## References

[1] Russell NJ, Seale AC, O’Driscoll M, O’Sullivan C, Bianchi-Jassir F, Gonzalez-Guarin J, et al. Maternal colonization with Group B Streptococcus and serotype distribution worldwide: systematic review and meta-analyses. Clin Infect Dis. 2017;65(suppl_2):S100–11. doi: 10.1093/cid/cix658 29117327 PMC5848259

[2] Madrid L, Seale AC, Kohli-Lynch M, Edmond KM, Lawn JE, Heath PT, et al. Infant group B streptococcal disease incidence and serotypes worldwide: systematic review and meta-analyses. Clin Infect Dis. 2017;65(suppl_2):S160–72. 10.1093/cid/cix656 PMC5850457 29117326

[3] Gonçalves BP, Procter SR, Paul P, Chandna J, Lewin A, Seedat F, et al. Group B streptococcus infection during pregnancy and infancy: estimates of regional and global burden. Lancet Glob Health. 2022;10(6):e807–19. doi: 10.1016/S2214-109X(22)00093-6 35490693 PMC9090904

[4] Seale AC, Bianchi-Jassir F, Russell NJ, Kohli-Lynch M, Tann CJ, Hall J, et al. Estimates of the burden of Group B Streptococcal disease worldwide for pregnant women, stillbirths, and children. Clin Infect Dis. 2017;65(suppl_2):S200–19. doi: 10.1093/cid/cix664 29117332 PMC5849940

[5] Kwatra G, Izu A, Cutland C, Akaba G, Ali MM, Ahmed Z, et al. Prevalence of group B Streptococcus colonisation in mother-newborn dyads in low-income and middle-income south Asian and African countries: a prospective, observational study. Lancet Microbe. 2024;5(10):100897. doi: 10.1016/S2666-5247(24)00129-0 39178870 PMC11464403

[6] Le Doare K, O’Driscoll M, Turner K, Seedat F, Russell NJ, Seale AC, et al. Intrapartum Antibiotic Chemoprophylaxis Policies for the Prevention of Group B Streptococcal Disease Worldwide: Systematic Review. Clin Infect Dis. 2017;65(suppl_2):S143–s51. 10.1093/cid/cix654 PMC5850619 29117324

[7] Organization WH. Group B streptococcus vaccine: full value of vaccine assessment: policy and implementation issues. 2021.

[8] Black RE, Victora CG, Walker SP, Bhutta ZA, Christian P, de Onis M, et al. Maternal and child undernutrition and overweight in low-income and middle-income countries. Lancet. 2013;382(9890):427–51. doi: 10.1016/S0140-6736(13)60937-X 23746772

[9] Fischer Walker C, Black RE. Zinc and the risk for infectious disease. Annu Rev Nutr. 2004;24:255–75. doi: 10.1146/annurev.nutr.23.011702.073054 15189121

[10] Wu C, Labrie J, Tremblay YDN, Haine D, Mourez M, Jacques M. Zinc as an agent for the prevention of biofilm formation by pathogenic bacteria. J Appl Microbiol. 2013;115(1):30–40. doi: 10.1111/jam.12197 23509865

[11] Buzza KM, Pluen A, Doherty C, Cheesapcharoen T, Singh G, Ledder RG, et al. Modulation of biofilm formation and permeability in Streptococcus mutans during exposure to zinc acetate. Microbiol Spectr. 2023;11(2):e0252722. doi: 10.1128/spectrum.02527-22 36809043 PMC10100724

[12] Gaddy JA, Radin JN, Cullen TW, Chazin WJ, Skaar EP, Trent MS, et al. Helicobacter pylori resists the antimicrobial activity of calprotectin via Lipid A modification and associated biofilm formation. mBio. 2015;6(6):e01349–15. doi: 10.1128/mBio.01349-15 26646009 PMC4669380

[13] Subramanian Vignesh K, Deepe GS Jr. Immunological orchestration of zinc homeostasis: The battle between host mechanisms and pathogen defenses. Arch Biochem Biophys. 2016;611:66–78. doi: 10.1016/j.abb.2016.02.020 26921502 PMC4996772

[14] Miyoshi Y, Tanabe S, Suzuki T. Cellular zinc is required for intestinal epithelial barrier maintenance via the regulation of claudin-3 and occludin expression. Am J Physiol Gastrointest Liver Physiol. 2016;311(1):G105–16. doi: 10.1152/ajpgi.00405.2015 27151944

[15] Francis JD, Guevara MA, Lu J, Madhi SA, Kwatra G, Aronoff DM, et al. The antimicrobial activity of zinc against group B Streptococcus is strain-dependent across diverse sequence types, capsular serotypes, and invasive versus colonizing isolates. BMC Microbiol. 2022;22(1):23. doi: 10.1186/s12866-021-02428-3 35026981 PMC8756620

[16] Kwatra G, Adrian PV, Shiri T, Buchmann EJ, Cutland CL, Madhi SA. Serotype-specific acquisition and loss of group B streptococcus recto-vaginal colonization in late pregnancy. PLoS One. 2014;9(6):e98778. doi: 10.1371/journal.pone.0098778 24979575 PMC4076185

[17] Tanvir EM, Komarova T, Comino E, Sumner R, Whitfield KM, Shaw PN. Effects of storage conditions on the stability and distribution of clinical trace elements in whole blood and plasma: application of ICP-MS. J Trace Elem Med Biol. 2021;68:126804. doi: 10.1016/j.jtemb.2021.126804 34111708

[18] Gibson RS, Hess SY, Hotz C, Brown KH. Indicators of zinc status at the population level: a review of the evidence. Br J Nutr. 2008;99 Suppl 3:S14–23. doi: 10.1017/S0007114508006818 18598584

[19] Lassi ZS, Haider BA, Bhutta ZA. Zinc supplementation for the prevention of pneumonia in children aged 2 months to 59 months. Cochrane Database Syst Rev. 2010;(12):CD005978. doi: 10.1002/14651858.CD005978.pub2 21154362

[20] Osendarp SJ, van Raaij JM, Darmstadt GL, Baqui AH, Hautvast JG, Fuchs GJ. Zinc supplementation during pregnancy and effects on growth and morbidity in low birthweight infants: a randomised placebo controlled trial. Lancet. 2001;357(9262):1080–5. doi: 10.1016/s0140-6736(00)04260-4 11297959

[21] Sambu W. Child nutrition. South African Child Gauge. 2020;2020.

[22] Hotz C, Peerson JM, Brown KH. Suggested lower cutoffs of serum zinc concentrations for assessing zinc status: reanalysis of the second National Health and Nutrition Examination Survey data (1976-1980). Am J Clin Nutr. 2003;78(4):756–64. doi: 10.1093/ajcn/78.4.756 14522734

[23] Harika R, Faber M, Samuel F, Kimiywe J, Mulugeta A, Eilander A. Micronutrient status and dietary intake of iron, vitamin a, iodine, folate and zinc in women of reproductive age and pregnant women in Ethiopia, Kenya, Nigeria and South Africa: a systematic review of data from 2005 to 2015. Nutrients. 2017;9(10):1096. doi: 10.3390/nu9101096 28981457 PMC5691713

[24] Stevens GA, Beal T, Mbuya MNN, Luo H, Neufeld LM, Global Micronutrient Deficiencies Research Group. Micronutrient deficiencies among preschool-aged children and women of reproductive age worldwide: a pooled analysis of individual-level data from population-representative surveys. Lancet Glob Health. 2022;10(11):e1590–9. doi: 10.1016/S2214-109X(22)00367-9 36240826 PMC10918648

[25] Nishihara Y, Dangor Z, French N, Madhi S, Heyderman R. Challenges in reducing group B Streptococcus disease in African settings. Arch Dis Child. 2017;102(1):72–7. doi: 10.1136/archdischild-2016-311419 27831912 PMC5256401

[26] Gibson RS, Hess SY, Hotz C, Brown KH. Indicators of zinc status at the population level: a review of the evidence. Br J Nutr. 2008;99 Suppl 3:S14–23. doi: 10.1017/S0007114508006818 18598584

[27] Hess SY, Peerson JM, King JC, Brown KH. Use of serum zinc concentration as an indicator of population zinc status. Food Nutr Bull. 2007;28(3 Suppl):S403–29. doi: 10.1177/15648265070283S303 17988005

