# Supplementary material for: Higher serum zinc micronutrient levels are associated with reduced susceptibility to Group B Streptococcus rectovaginal colonisation in pregnant women
Source: PLoS One. 2026 Mar 12;21(3):e0344689. doi: 10.1371/journal.pone.0344689 (PMC12981460; doi:10.1371/journal.pone.0344689)
Supplement: S1 File — (DOCX) [file pone.0344689.s001.docx]

**S1 Table: Baseline demographic and clinical characteristics of pregnant women**

| **Variable** | **New Acquisition**  **(n=81)** | **Persistently un-colonised**  **(n=242)** | **P value** |
| --- | --- | --- | --- |
| Mean age; years ± SD | 25.81 ± 5.11 | 25.96 ± 5.74 | 0.98^#^ |
| Mean gestational age at visit-1; weeks ± SD^§^ | 22.86 ± 2.32 | 22.71 ± 2.11 | 0.65^#^ |
| Gravidity; median [IQR] | 2; [1, 2] | 2; [1, 2] | 0.25^##^ |
| Parity; median [IQR] | 0; [0, 1] | 0; [0, 1] | 0.87^##^ |
|  | **Colonisation clearance (n=76)** | **Persistently colonised**  **(n=67)** |  |
| Mean age; years ± SD | 26.05 ± 5.15 | 26.85 ± 6.38 | 0.52^###^ |
| Mean gestational age at visit-1; weeks ± SD^§^ | 23.03 ± 2.32 | 22.83 ± 2.22 | 0.54^#^ |
| Gravidity; median [IQR] | 2; [1, 3] | 2; [1, 3] | 0.96^##^ |
| Parity; median [IQR] | 1; [0, 1] | 1; [0, 1] | 0.90^##^ |

Abbreviations: IQR: interquartile range; SD: standard deviation

^#^ P value derived using Mann Whitney test, ^##^ P value derived using logistic regression, ^###^ P value derived using student unpaired t-test, §Based on 80 new acquisition cases and 66 persistently colonised cases for which gestational age was available

**
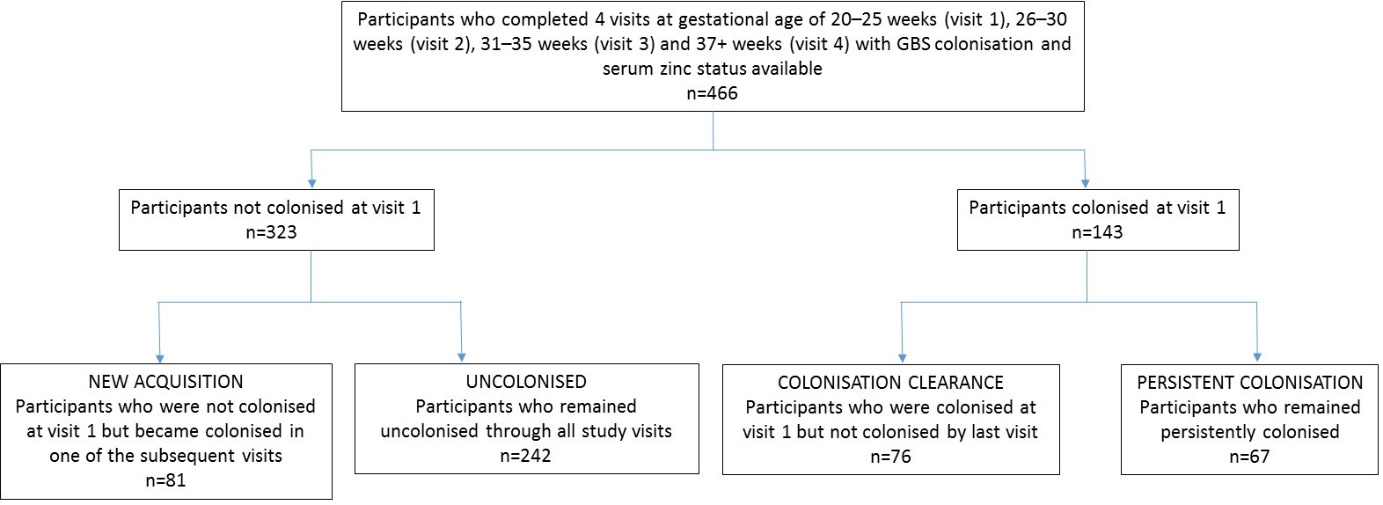
**

**S1 Figure: Eligible participants for the study**

**S2 Figure: Serum zinc concentrations of women at visit-1 and visit-4.**

Represented are visits on X axis and zinc concentrations (grey dots) in µmol/L (in log base 10) on Y axis, GMC (thick solid line), 95%CI (thin solid line), *p value. Visit-1 (n=466) zinc GMC (18.32 µmol/L, 95% CI 16.98-19.77); Visit-4 (n=464) zinc GMC (20.63 µmol/L, 95% CI 18.86-22.57). Zinc GMCs at visit 4 was based on 464 participants due to unavailability of visit 4 zinc status for 2 participants.


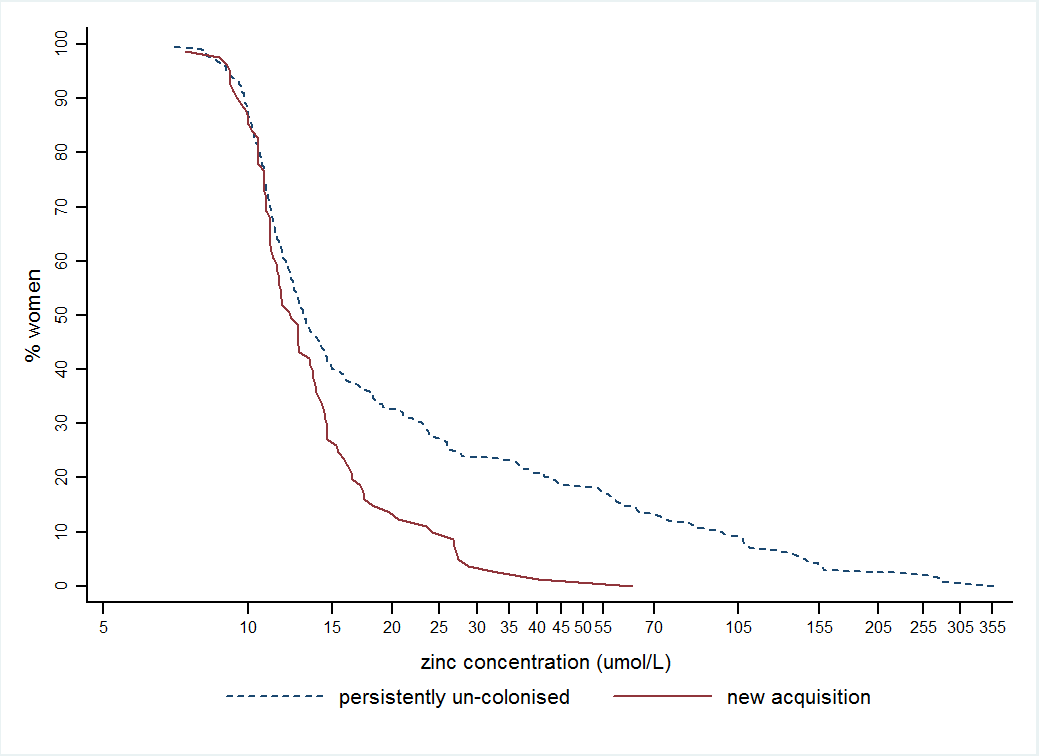


**S3 Figure: Reverse cumulative plots of zinc concentrations of pregnant women who had GBS new acquisition and those who remained persistently un-colonised with GBS.**


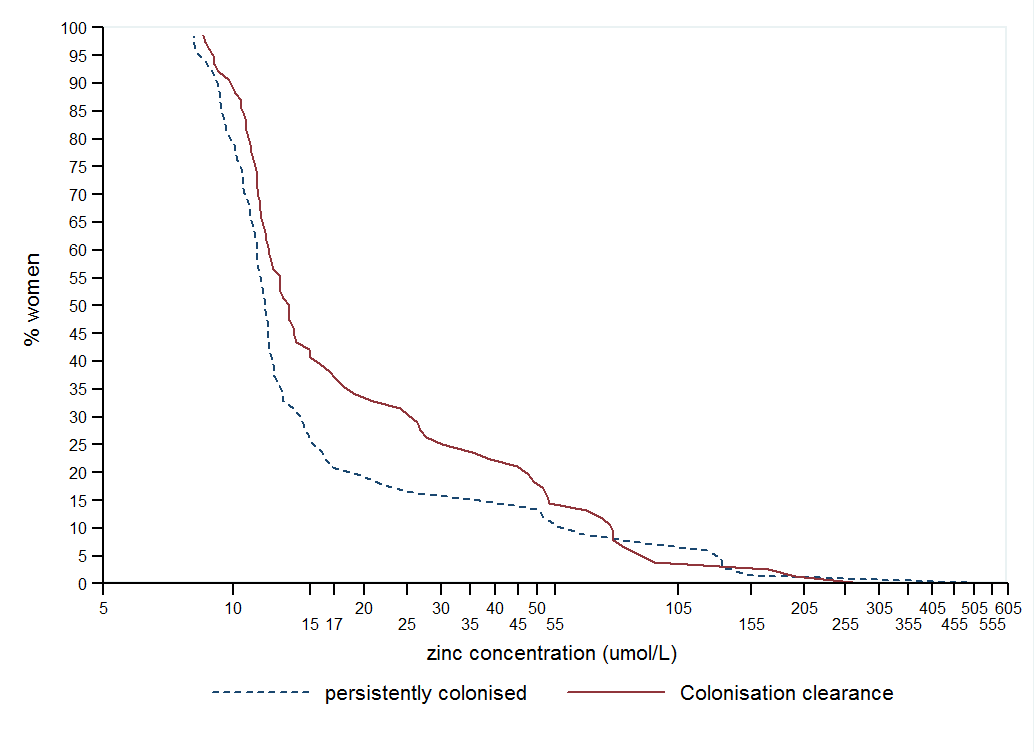


**S4 Figure: Reverse cumulative plots of zinc concentrations of pregnant women who cleared GBS colonisation and those who remained persistently colonised with GBS.**
